# A Method of Easily Effecting the Solution of Salvarsan and a Comparison of the Value of Salvarsan and Neo-Salvarsan

**Published:** 1914-01

**Authors:** John Banks

**Affiliations:** LaGrange, Ga.


### Journal-Record of Medicine

Successor to Atlanta Medical and Surgical Journal, Established 1855
and Southern Medical Record, Established 1870

**OWNED BY THE ATLANTA MEDICAL JOURNAL COMPANY**

PUBLISHED MONTHLY

*Official Organ Fulton County Medical Society, State Examining Board, Presbyterian Hospital, Atlanta, Birmingham and Atlantic Railroad Surgeons' Association, Chattahoochee Valley Medical and Surgical Association, Etc.*

EDGAR BALLENGER, M. D., Editor
BERNARD WOLFF, M. D., Supervising Editor
A. W. STIRLING, M. D., C. M., D. P. H.; J. S. HURT, B. Ph., M.D.
GEO. M. NILES, M. D., W. J. LOVE, M. D., (Ala.); Associate Editors
E. W. ALLEN, Business Manager

**COLLABORATORS**

DR. W. F. WESTMORLAND, General Surgery
F. W. McRAE, M. D., Abdominal Surgery
H. F. HARRIS, M. D., Pathology and Bacteriology
E. B. BLOCK, M. D., Diseases of the Nervous System
MICHAEL HOKE, M. D., Orthopedic Surgery
CYRUS W. STRICKLER, M. D., Legal Medicine and Medical Legislation
E. C. DAVIS, A. B., M. D., Obstetrics
E. G. JONES, A. B., M. D., Gynecology
R. T. DORSEY, JR., B. S., M. D., Medicine
L. M. GAINES, A. B., M. D., Internal Medicine
GEO. C. MIZELL, M. D., Diseases of the Stomach and Intestines
L. B. CLARKE, M. D., Pediatrics
EDGAR PAULIN, M. D., Opsonic Medicine
THEODORE TOEPEL, M. D., Mechano Therapy
R. R. DALY, M. D., Medical Society
A. W. STIRLING, M. D., Etc., Diseases of the Eye, Ear, Nose and Throat
BERNARD WOLFF, M. D., Diseases of the Skin
E. G. BALLENGER, M. D., Diseases of the Genito-Urinary Organs

VOL. LX. ATLANTA, GA., JANUARY, 1914. NO. 10

### A METHOD OF EASILY EFFECTING THE SOLUTION OF SALVARSAN AND A COMPARISON OF THE VALUE OF SALVARSAN AND NEO-SALVARSAN.

BY JOHN BANKS, A. B., M. D., LAGRANGE, GA.

So much has been written concerning the administration of salvarsan that any addition to the literature must have a well founded reason.

The question of the suspected superiority of the old salvarsan over neo-salvarsan, which superiority one is tempted to sacrifice for the ease in mixing the latter, is my excuse for submitting a method of preparing salvarsan which will enable any one to get the drug in solution in a few moments with the assurance that it is as nearly neutral as it is possible to make it. The ease with which the solution is accomplished leaves no excuse for the use of neosalvarsan if it be true, as a number of eminent syphylographers contend, that the original product is more efficacious than the later modified preparation.

The reagents required are a 15% or 20% solution of NaOH; a 3% to 5% solution of HCl and sterile solution of N salt. A small glass mortar and pestle and two eye-droppers complete the apparatus. Note that the method does not require any very definite or accurate percentage of the reagents used; an important consideration where the average drug clerk has to be depended upon for supplying same. It would be highly important that the solution of NaOH is exactly of the required 15% if the directions given in the circular accompanying the dose of Salvarsan are followed, since where a given number (19) of drops of NaOH are used as per directions, any inaccuracy in the strength of the NaOH would result either in insufficient NaOH to dissolve the drug if the solution is too weak, or an excess of alkali if it is too strong.

The ampoule of salvarsan is emptied into the mortar and twenty drops of the NaOH solution added from the eye-dropper. It is not necessary to be careful about the number of drops used. Next about one-half ounce of the N-1 salt solution is added and the whole stirred and rubbed until a perfectly clear solution is effected. In case the drug does not readily go into solution, more NaOH is added until it does, nor is it necessary that any exact record of the number of drops of NaOH used be kept.

So far, the above is similar to the procedure advised in the direction accompanying the drug except that no definite number of drops or strength of NaOH is required. My experience in mixing salvarsan by the directions of the manufacturers has

).

been that it is difficult to obtain a solution of NaOH from the drug stores of ACCURATE 15% strength and that the solution so obtained will rarely effect a clear solution of the salvarsan when the ordered nineteen drops is added. In case it becomes necessary to add more NaOH than the stated number of drops, the operator is at a loss to know whether the mixture does or does not contain a harmful excess of alkali.

Having obtained a perfectly clear solution by the above procedure which is known to contain a considerable excess of NaOH, we proceed by means of the 5% solution of HCl to throw the solution back to acid. The HCl is added drop by drop until the mixture turns cloudy and of about the consistency of thin cream with much the same appearance. This is thoroughly stirred and rubbed until it is perfectly free from lumps and of a smooth, opaque, creamy appearance. Now comes the final addition of NaOH drop by drop, which is the only part of the procedure that calls for any exactness and care. The NaOH is added a drop at a time, stirring constantly, during which time it will be noted that the mixture gradually grows clearer with the addition of each drop of the soda. Finally a point is reached at which the addition of one more drop of NaOH will change the slightly cloudy solution, in a flash, from cloudy to perfectly clear, at which point the addition of NaOH is stopped.

It is obvious that the mixture being opaque and then being made perfectly clear by the addition of the final drop of soda, we have effected the clear solution of the salvarsan with the least possible amount of free or excess alkali.

So nicely poised on the line betwen acid and alkali can the solution be made by the above procedure that it will sometimes be found to again turn cloudy when the final volume of N-1 salt is added to dilute it up to the required amount for injection. This is caused by the slight trace of free HCl in the salt solution which is sufficient to throw the delicate balance back over the acid line. When this occurs, the addition of from one to two drops of NaOH to the diluted dose will immediately clear it up.

The above sounds much more complicated in the describing than it is in practice. It must be remembered that at no stage of the procedure is it required to observe any accuracy of measurement except when titrating the final milky mixture with the last few drops of NaOH required to effect a clear solution.

The excess of NaOH permissable in the initial mixing effects an almost instantaneous solution of the Salvarsan and a perfectly smooth and homogeneous product results from the further addition of HCl solution. When this point has been reached it is only required that the NaOH be added slowly and with constant stirring with the pestle so that the final flash from cloudy to clear will be obtained by one final drop of NaOH.

The end result of the mixing of NaOH with HCl is NaCl which does not affect the original value of the Normal Salt.

It may be of interest in this connection and in view of the claim that severe reactions follow the use of water not freshly distilled, to state that the writer has used the above method of preparing salvarsan in over one hundred cases and not only has not been careful to use freshly distilled water but has used only freshly boiled tap water from the city supply that had not been distilled at all. No alarming symptoms have been noted in any case. A slight chill followed by a temperature not exceeding 102 F., and, in a few instances, mild nausea, have been the most pronounced after effects.

In view of the above is it not reasonable to suggest that marked toxic effects may have been due to a great and unnecessary excess of NaOH in mixing the dose, which might easily come about from following the printed directions with a measured 19 drops of a 15% solution of NaOH prepared in the drug store without accurate weighing of the sodium hydroxid to insure a solution that is exactly 15%?

By the method explained, there is no chance of an excess of alkali in the final solution and I am disposed to attribute the freedom from marked post-operative symptoms in my cases, to the accuracy of the neutralization secured by this method.

I have found, moreover, that just as marked symptoms sometimes follow the use of neosalvarsan as of salvarsan when prepared by the above titration emthod, and can see no advantage in the use of neosalvarsan when the solution of salvarsan can be so readily effected, and particularly am I loath to give up the old for the new when it is, in the opinion of many, at a sacrifice of the superior curative properties of the latter.

Since the above was written an opportunity has been afforded me through the courtesy of Dr. H. R. Slack, by whose permission I am reporting the following, to make an interesting comparison of the two drugs.

CASE 1. Miss Fannie H.; white, 18 yrs. Initial lesion of the lip. Began two months ago following the smoking of a cigarette "butt" left by her brother who is known to be luetic. At time of injection presented typical syphilitic ulcer on lip with diffuse copper colored syphylide covering face, neck and arms. Mucous patches in mouth and sore throat.

CASE 2. Miss Bessie H.; white, 16 yrs. First cousin of Case 1 and in identical condition. Same source and manner of infection with same location of initial lesion. Secondary symptoms in exactly the same stage of progression. Here we had two girls, first cousins, with an infection from the same source and therefore of the same virulence in precisely the same stage. The opportunity was too tempting to forego, accordingly they were given, Case 1, a full dose (0.60 grams) intravenously of salvarsan; Case 2, a full dose (0.90 grams) intravenous, of neosalvarsan.

The result five days later was so marked that Case 2 jealously inquired, "Why hasn't the breaking out on my face gone like it has on Fannie's?" At this time Case 1 showed a complete absence of all symptoms. The skin was entirely cleared up, the ulcer of the lip was decidedly improved; healthy, red and beginning to granulate over; sore throat and mucous patches well.

Case 2 showed decided improvement. The ulcer of the lip was healthy looking and had decreased in size though not so markedly as in 1. The sore throat was improved but some mucous patches still present. The syphilides in the two cases showed the most marked contract and since their "complexion"

was, to them, the matter of most import, the disappointment of case 2 was proportionately acute, whereas the skin in Case 1 was perfectly normal and of a smooth, clear pink without a vestige of the syphilide, Case 2 had begun to clear up but slightly. Since that time (two weeks later), Case 2 has still further improved, though there is still a noticeable "stain" at the site of the old skin lesions. The ulcer on the lip is not yet entirely healed. Case 1 has no vestige of any symptom remaining. No Wassermann was made. These cases will both receive another injection next week and, needless to say, this time both will get salvarsan.

